# Chemotherapy‐induced peripheral neuropathy in African American cancer survivors: Risk factors and quality of life outcomes

**DOI:** 10.1002/cam4.4328

**Published:** 2021-10-23

**Authors:** Matthew R. Trendowski, Christine M. Lusk, Julie J. Ruterbusch, Randell Seaton, Michael S. Simon, Mark K. Greenwald, Felicity W. K. Harper, Jennifer L. Beebe‐Dimmer, Ann G. Schwartz

**Affiliations:** ^1^ Wayne State University School of Medicine Department of Oncology Detroit Michigan USA; ^2^ Karmanos Cancer Institute Detroit Michigan USA; ^3^ Wayne State University School of Medicine Department of Psychiatry and Behavioral Neurosciences Detroit Michigan USA

**Keywords:** African Americans, chemotherapy‐induced peripheral neuropathy, health disparities, quality of life, risk

## Abstract

**Background:**

Epidemiological studies of chemotherapy‐induced peripheral neuropathy (CIPN) have predominantly focused on non‐Hispanic White patients, despite the observation that African Americans are more likely to experience CIPN. To address this health disparities gap, we sought to identify non‐genetic risk factors and comorbidities associated with CIPN in African American cancer survivors using the Detroit Research on Cancer Survivors study.

**Methods:**

Logistic regression was used to evaluate relationships between presence of self‐reported CIPN and relevant clinical characteristics in 1045 chemotherapy‐treated African American cancer survivors. Linear regression was used to evaluate risk factors for CIPN and quality of life outcomes that reflect physical, social, emotional, and functional domains of health.

**Results:**

Patients with CIPN were more likely to report hypertension (OR = 1.28, 95% CI: 0.98–1.67, *p* = 0.07), hypercholesterolemia (OR = 1.32, 95% CI: 1.001–1.73, *p* = 0.05), history of depression (OR = 1.62, 95% CI: 1.18–2.25, *p* = 0.003), and diabetes (OR = 1.33, 95% CI: 0.98–1.82, *p* = 0.06) after adjustment for age at diagnosis, sex, and cancer site. BMI (OR = 1.02 kg/m^2^, 95% CI: 1.006–1.04 kg/m^2^, *p* = 0.008) was also positively associated with CIPN. In addition, CIPN status was significantly associated with quality of life (FACT‐G total: *β* = −8.60, 95% CI: −10.88, −6.32) *p* < 0.0001) and mood (PROMIS^®^ Anxiety: *β* = 4.18, 95% CI: 2.92–5.45, *p* < 0.0001; PROMIS^®^ Depression: *β* = 2.69, 95% CI: 1.53–3.84, *p* < 0.0001) after adjustment for age at diagnosis, sex, cancer site, and comorbidities. Neither alcohol consumption (OR = 0.88, 95% CI: 0.68–1.14, *p* = 0.32) nor tobacco use (ever smoked: OR = 1.04, 95% CI: 0.80–1.35, *p* = 0.76; currently smoke: OR = 1.28, 95% CI: 0.90–1.82, *p* = 0.18) was associated with increased CIPN risk.

**Conclusion:**

Risk factor profiles in African Americans are not entirely consistent with those previously reported for non‐Hispanic White patients. Neglecting to understand the correlates of common chemotherapy‐induced toxicities for this patient population may further contribute to the health disparities these individuals face in receiving adequate healthcare.

## Graphical abstract

Although African Americans have an increased susceptibility to developing chemotherapy‐induced peripheral neuropathy, epidemiological studies have predominantly focused on non‐Hispanic Whites. Our study demonstrates that risk factor profiles in African Americans are not entirely consistent with those previously reported for non‐Hispanic Whites, and that neglecting to understand the correlates of common chemotherapy‐induced toxicities for this patient population may further contribute to the health disparities these individuals face in receiving adequate healthcare.

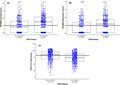

## 1 INTRODUCTION

Advances in chemotherapy have markedly improved overall cancer survival, enabling more patients to live decades after completing therapy.^1^ However, many survivors experience persistent off‐target toxicities due to chemotherapy. This is epitomized by chemotherapy‐induced peripheral neuropathy (CIPN), which manifests as a tingling, numbness, weakness, or burning pain in the extremities, which is a common persistent adverse event in adult‐onset cancer survivors. A meta‐analysis of 31 studies evaluating the long‐term effects of CIPN found that approximately 30.0% of cancer survivors have persistent symptoms 6 months or longer after treatment has been completed.^2^ Several agents appear to greatly increase susceptibility to persistent toxicity, as 56.2% of testicular cancer survivors reported CIPN 5 years after treatment with cisplatin‐based chemotherapy,^3^ while 41% of breast cancer survivors reported CIPN 3 years after initiating paclitaxel‐based chemotherapy.^4^

Due to its prevalence among long‐term cancer survivors, multiple studies have attempted to identify demographic, clinical, and behavioral risk factors and comorbidities associated with CIPN, with associations being identified for age, smoking status, alcohol use, hypertension, and BMI/weight gain.^2^, ^3^, ^5^, ^6^ However, these studies have predominantly focused on non‐Hispanic White patients, despite the observation that CIPN prevalence varies across patient populations. Notably, African American patients have a markedly increased risk for both moderate (grades 2–4) and severe CIPN.^7^ Lack of patient diversity in long‐term toxicity studies is problematic because non‐genetic risk factors and comorbidities in European populations may not be applicable in underserved communities, creating a health disparities gap. Therefore, previously identified associations with CIPN need to be explored in non‐European patients to ensure their validity across patient populations, as does the impact of persistent toxicity on the overall quality of life. With high overall 5‐year survival rates for several cancers commonly treated with CIPN‐inducing agents,^8^, ^9^ survivors who develop CIPN may endure the consequences for decades; therefore, understanding how CIPN affects quality of life is essential.

The Detroit Research on Cancer Survivors (ROCS) study is a longitudinal study of African American cancer survivors in the Metropolitan Detroit area. It is currently the largest dataset of African American cancer survivors and examines the overall quality of life using physical, social, emotional, and functional measures, making it an excellent resource for analyzing the effects of persistent chemotherapy‐induced toxicities in African American patients. In this study, we use the Detroit ROCS dataset to examine whether demographic and clinical factors contribute to CIPN risk in patients of African ancestry, and whether CIPN is associated with several quality of life measures.

## 2 METHODS

### 2.1 Patient selection

All patients were enrolled in Detroit ROCS, a cohort of self‐identified African American cancer survivors in the Metropolitan Detroit area.^10^ Eligible cancer survivors are defined as those with primary breast, colorectal, lung, or prostate cancer diagnosed after 1 January 2013, or endometrial cancer or any other cancer diagnosed before age 50 (“young onset”) after 1 January 2016. Cases were identified through the Metropolitan Detroit Cancer Surveillance System (MDCSS), a population‐based cancer registry covering metropolitan Detroit and a founding participant in NCI’s Surveillance, Epidemiology, and End Results program. Recruitment for Detroit ROCS is ongoing and planned through the end of 2021 with the goal of enrolling 5000 survivors. Results presented here include data from the first 1045 ROCS participants reporting chemotherapy treatment. The Karmanos Cancer Institute Protocol and Monitoring Review Board and the Wayne State University Institutional Review Board (#050417M1F) reviewed and approved this research with all participants in the Detroit ROCS study providing informed consent.

### 2.2 Patient outcomes measurement

Participants completed a survey that assessed sociodemographic factors and financial hardship, medical history and medication use, family history of cancer, health behaviors (tobacco and alcohol use, diet, and physical activity), cancer treatment history, and cancer screening practices. Surveys were completed online via Qualtrics, using a mailed survey, or over the phone with a trained interviewer. Cancer‐related information including cancer site, stage, and time since diagnosis was obtained via linkage with MDCSS.

To establish the presence of CIPN, patients were first asked, “Have you ever had chemotherapy for your cancer (oral or IV)?” Patients who responded “Yes” were then asked, “Since receiving chemotherapy have you ever experienced numbness, pain, or tingling in your hands or feet?” All patients who reported having chemotherapy and responded to the CIPN question were included in the analysis (*n* = 1045). Patients who reported continued symptoms at the time of the survey were designated as having CIPN and were used as cases in the study. To evaluate whether CIPN severity was associated with quality of life measures, participants who met these criteria were also asked to describe the degree of numbness, pain, or tingling in their hands or feet with the following options: mild and does not interfere with activities of daily living (1), moderate and does not interfere with activities of daily living (2), moderate to severe and interferes with activities of daily living (3), or severe and completely prevents most activities of daily living (4).

Patients completed questionnaires assessing adverse events, lifestyle habits, comorbidities, prior therapy, and medication use, as previously described.^10^ In addition to chemotherapy, patients were asked about whether their cancer was treated with other treatment modalities, including surgery, hormone therapy, immunotherapy, and radiotherapy. Self‐reported history of depression,^11^ diabetes,^12^ high cholesterol,^3^ and hypertension^3^ at the time of ROCS enrollment were chosen as clinical characteristics to be evaluated against CIPN status because they have previously been associated with peripheral neuropathy. All patients were evaluated by the following question: “Has a doctor ever told you that you have any of the following medical conditions?” Total comorbidity count was evaluated to examine whether CIPN status was associated with other negative health outcomes, and included the following health conditions: obesity (evaluated by BMI reported at study entry), myocardial infarction, coronary heart failure, atrial fibrillation, coronary artery disease, stroke, hypertension, high cholesterol, chronic obstructive pulmonary disease (COPD), emphysema, hepatitis, arthritis, diabetes, fracture over age 50, thyroid problem, and depression. COPD and/or emphysema were counted as 1 summary comorbidity, while any heart problems (atrial fibrillation, coronary artery disease, coronary heart failure, or myocardial infarction) were counted as 1 summary comorbidity.

Health behaviors assessed included physical activity, alcohol consumption, and smoking status. Physical activity was defined in accordance with the International Physical Activity Questionnaire.^13^ Any physical activity was defined as participating in any activity that either improves fitness or increases heart rate (e.g., jogging and yard work). Moderate physical activity was defined as causing small increases in breathing or heart rate (e.g., walking briskly, biking on level ground or with few hills, and playing golf). Vigorous physical activity was defined as large increases in breathing or heart rate, during which an individual can only say a few words without stopping to catch their breath (e.g., aerobics, jogging or running, and swimming laps). Alcohol consumption was assessed qualitatively as consuming at least one alcoholic beverage in the 4 weeks prior to survey completion and quantitatively as number of standard drinks per week (beer, liquor, malt beverage, and/or wine). Patients who consumed between 0 and 4 drinks per week were grouped separately, while those who drank five or more drinks per week were grouped together due to small sample size. Smoking was categorized as ever, never, and current based on survey responses to tobacco use questions. In addition, smoking was evaluated by number of cigarettes smoked per day categorized as: none, less than a half a pack per day (1–9 cigarettes), between half a pack and less than a pack (10–19 cigarettes), and a pack or more per day (≥20 cigarettes).

Sociodemographic characteristics were also evaluated for statistical association with CIPN. Poverty level was assessed using the census tract poverty indicator, a metric that categorizes economic security based on the poverty rate, the percentage of the population in a census‐tract classified as being below the official poverty threshold according to the 2005–2009 American Community Survey while also taking into account family size and age composition (the number of children under 18) and inflation, as previously described.^14^ Patients were grouped into the following categories: 0%–<5% poverty (1); 5%–<10% poverty (2); 10%–<20% poverty (3); and 20%–100% poverty (4). Financial income was also directly assessed by asking patients about their income in the year prior to the survey, before taxes. Responses were grouped as follows: <$20,000 (1); $20,000–$39,999 (2); $40,000–$59,999 (3); $60,000–$79,999 (4); and ≥$80,000 (5).

Several measures were used to evaluate patients' quality of life following chemotherapy. The Functional Assessment of Cancer Therapy‐General (FACT‐G),^15^ includes four subscales: physical well‐being (PWB), social/family well‐being (SWB), emotional well‐being (EWB), and functional well‐being (FWB). Each subscale includes six to seven statements (e.g., “I have a lack of energy”) and participants were asked to rate the extent to which each statement applied to them in the past 7 days using a five‐point scale (0 = “not at all” to 4 = “very much”). The reliability and validity of the FACT‐G and the site‐specific measures have been extensively documented, with a two‐point difference in the subscale scores and a 5‐point difference in the total FACT‐G score being associated with meaningful differences on clinical and subjective indicators.^16^ Specifically, in elderly patients with cancer, the mean total FACT‐G score reported in the literature is 82.2 ± 16.2 SD^17^ with lower scores indicating poorer quality of life. Patient‐Reported Outcomes Measurement Information System (PROMIS^®^), a multi‐step, mixed methods approach to assessing physical, emotional, and social health^18^ was used to evaluate emotional health. Using the PROMIS^®^ Anxiety 4a and Depression 4a measures, patients were asked about their fear (e.g., worry and feelings of panic), anxious misery (e.g., dread), hyperarousal (e.g., tension, nervousness, and restlessness), and somatic symptoms related to arousal (e.g., cardiovascular symptoms and dizziness), while the PROMIS^®^ Depression measure assesses affective and cognitive components of depression.^19^ A *T*‐score of 50 for PROMIS^®^ Anxiety and Depression measures reflects the US general population mean with higher scores indicating a more negative outcome.

### 2.3 Statistical analysis

Logistic regression was used to investigate associations between CIPN status and relevant clinical characteristics. For the logistic regression analysis, age at diagnosis, sex, and cancer site were included as covariates. Age at diagnosis was selected *a* priori, while sex and cancer site were selected due to their association with CIPN. We also performed linear regression to determine whether quality of life and mood were associated with CIPN. Characteristics that were significantly associated with CIPN (age at diagnosis, sex, cancer site, BMI at time of enrollment, diabetes, hypertension, hypercholesterolemia, and total comorbidity count) were included as covariates in the final linear regression model (note: depression was excluded in the comorbidity count given its use as an outcome variable). These analyses were also performed by cancer site. Analyses of breast, endometrial, and prostate cancer did not include sex as a covariate. Analyses were performed in R 3.3.2, with statistical significance set at *p* < 0.05.

## 3 RESULTS

### 3.1 Cohort characteristics

Demographic and clinical characteristics for African American cancer survivors included in this study are provided in Table 1 and Table S1. Of the 1045 patients included in the cohort, 550 patients reported having CIPN, while 495 did not report having the toxicity. Median age at diagnosis for all patients was 57 years (range: 21–79 years), while median age at the time of the survey was 59 years (range: 25–84 years). The most common primary cancer diagnosis was breast cancer (*n* = 523; 50.0%). Accordingly, the majority of the patients in the study were female (*n* = 795; 76.1%).

**TABLE 1:** Clinical and sociodemographic characteristics for African American cancer survivors treated with chemotherapy by reported chemotherapy‐induced peripheral neuropathy status

Characteristic	All patients	CIPN: No	CIPN: Yes
*n*	1045	495	550
Sex			
Male	250 (23.9%)	140 (28.3%)	110 (20.0%)
Female	795 (76.1%)	355 (71.7%)	440 (80.0%)
Age at diagnosis (years)			
Median (range)	57 (21–79)	58 (21–79)	56 (23–79)
Under 50	308 (29.5%)	144 (29.1%)	164 (29.8%)
50+	737 (70.5%)	351 (70.9%)	386 (70.2%)
Time from diagnosis to survey (months)			
Median (range)	19 (2–84)	20 (2–84)	17 (2–82)
Cancer site			
Breast	523 (50.0%)	222 (44.8%)	301 (54.7%)
Colorectal	193 (18.5%)	75 (15.2%)	118 (21.5%)
Endometrial	45 (4.3%)	14 (2.8%)	31 (5.6%)
Lung	161 (15.4%)	114 (23.0%)	47 (8.5%)
Prostate	46 (4.4%)	32 (6.5%)	14 (2.5%)
Other^a^	77 (7.4%)	38 (7.7%)	39 (7.1%)
BMI at enrollment (kg/m^2^)^b^			
Underweight (<18.5)	22 (2.1%)	15 (3.1%)	7 (1.3%)
Normal weight (18.5–25)	205 (19.8%)	106 (21.6%)	99 (18.3%)
Overweight (25–30)	326 (31.6%)	170 (34.6%)	156 (28.8%)
Obese (>30)	480 (46.4%)	200 (40.7%)	280 (51.7%)

Abbreviation: BMI, body mass index; CIPN, chemotherapy‐induced peripheral neuropathy.

^a^ Other cancers were only included for individuals diagnosed before age 50.

^b^ Twelve patients did not report their BMI at enrollment.

### 3.2 Associations with risk factors and comorbidities

Cancer site was significantly associated with CIPN (Table S2), as was sex, with females being more likely to experience CIPN following treatment than males (OR = 1.57, 95% CI: 1.19–2.10, *p* = 0.001). To determine whether the sex difference in CIPN predisposition was due in part to the high proportion of breast cancer patients in the study population (*n* = 523; 50%), we evaluated whether sex was associated with CIPN in colorectal (*n* = 193) or lung cancer (*n* = 161) patients. For both cancer sites, sex was not associated with CIPN (colorectal cancer: OR = 1.28, 95% CI: 0.65–2.58, *p* = 0.48; lung cancer: OR = 0.999, 95% CI: 0.54–1.86, *p* = 0.997). Both surgical treatment of cancer (covariate‐adjusted OR = 1.08, 95% CI: 0.995–1.17, *p* = 0.07) and radiotherapy (covariate‐adjusted OR = 0.94, 95% CI: 0.89–1.01, *p* = 0.07) were marginally associated with CIPN status, while immunotherapy (covariate‐adjusted OR = 0.93, 95% CI: 0.83–1.04, *p* = 0.20) and hormone therapy (covariate‐adjusted OR = 0.96, 95% CI: 0.88–1.04, *p* = 0.28) were not statistically significant (Table 2). The number of additional therapies patients received was also not associated with CIPN (covariate‐adjusted OR = 0.91, 95% CI: 0.78–1.06, *p* = 0.22). CIPN was inversely associated with vigorous physical activity (covariate‐adjusted OR = 0.71, 95% CI: 0.54–0.94, *p* = 0.02).

**TABLE 2:** Association between chemotherapy‐induced peripheral neuropathy and selected characteristics of African American cancer survivors adjusted for age at diagnosis, sex, and cancer site

Clinical characteristic	OR (95% CI)	*p* value	Covariate‐adjusted OR (95% CI)	Covariate‐adjusted *p* value
Cancer treatment				
Surgery	1.16 (1.08, 1.24)	**<0.0001**	1.08 (0.995, 1.17)	0.07
Immunotherapy	0.90 (0.80, 1.002)	0.06	0.93 (0.83, 1.04)	0.20
Radiotherapy	0.95 (0.90, 1.01)	0.13	0.94 (0.89, 1.01)	0.07
Hormone therapy	0.998 (0.92, 1.08)	0.97	0.96 (0.88, 1.04)	0.28
Number of additional therapies	1.04 (0.91, 1.20)	0.57	0.91 (0.78, 1.06)	0.22
Tobacco use				
Ever smoked (100 cigarettes)	0.86 (0.68, 1.11)	0.26	1.04 (0.80, 1.35)	0.76
Current smoker	1.08 (0.77, 1.53)	0.64	1.28 (0.90, 1.82)	0.18
Cigarettes per day	0.88 (0.78, 0.999)	0.05	0.97 (0.86, 1.11)	0.68
Alcohol use				
Alcohol consumption	0.90 (0.70, 1.15)	0.39	0.88 (0.68, 1.14)	0.32
Drinks per day	0.97 (0.90, 1.04)	0.40	0.99 (0.91, 1.07)	0.73
Physical activity				
Any physical activity	1.11 (0.86, 1.43)	0.40	1.07 (0.83, 1.39)	0.60
Moderate physical activity	1.08 (0.85, 1.38)	0.53	1.04 (0.81, 1.33)	0.75
Vigorous physical activity	0.73 (0.56, 0.95)	**0.02**	0.71 (0.54, 0.94)	**0.02**
BMI at enrollment	1.03 (1.01, 1.05)	**0.0004**	1.02 (1.006, 1.04)	**0.008**
Comorbidities total count	1.12 (1.05, 1.20)	**0.0009**	1.15 (1.07, 1.24)	**0.0001**
Hypertension	1.22 (0.95, 1.56)	0.12	1.28 (0.98, 1.67)	0.07
Hypercholesterolemia	1.19 (0.92, 1.54)	0.19	1.32 (1.001, 1.73)	**0.05**
Depression	1.65 (1.21, 2.27)	**0.002**	1.62 (1.18, 2.25)	**0.003**
Diabetes	1.28 (0.95, 1.72)	0.11	1.33 (0.98, 1.82)	0.06
Census tract poverty indicator	0.99 (0.86, 1.14)	0.89	1.04 (0.91, 1.21)	0.54
Gross annual income	0.99 (0.90, 1.09)	0.86	0.96 (0.87, 1.05)	0.36

Note

Bold indicates *p* ≤ 0.05.

Covariates in the logistic regression analysis include age at diagnosis, sex, and cancer site.

Abbreviation: BMI, body mass index.

**FIGURE 1:**
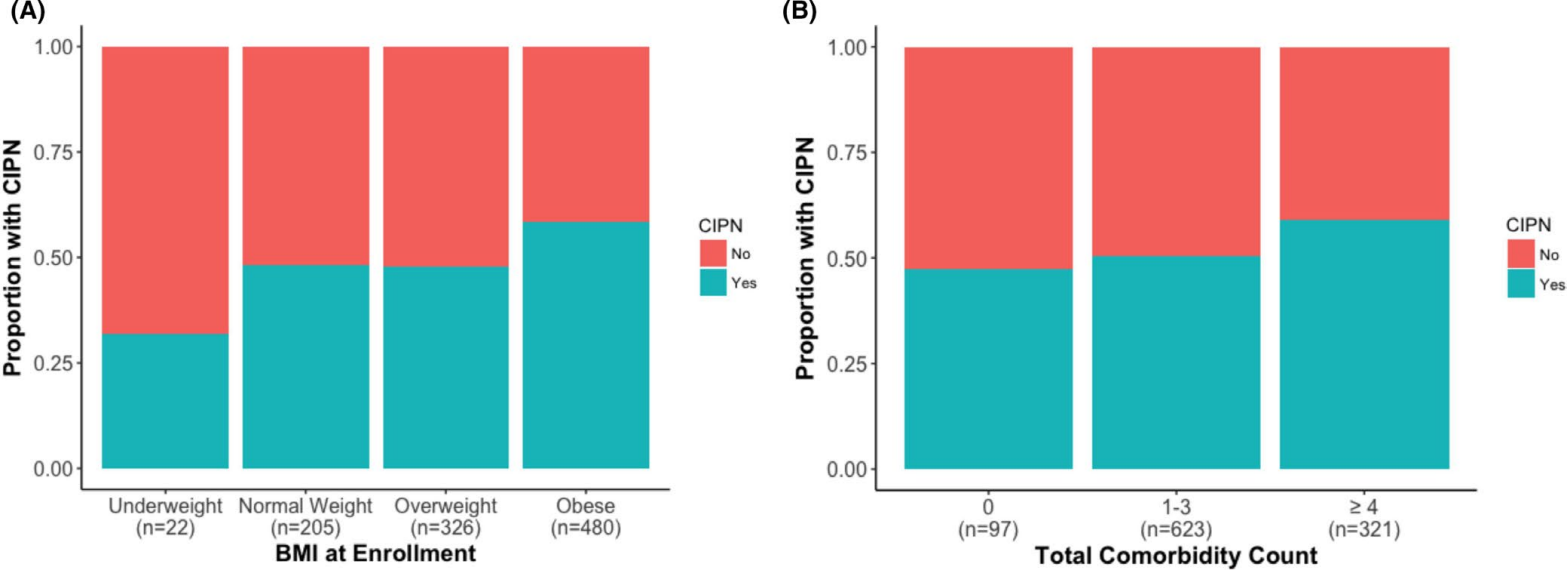
Distribution of body mass index (BMI) at enrollment and total comorbidity count in African American cancer survivors based on chemotherapy‐induced peripheral neuropathy (CIPN) status. The overall distribution of (A) BMI and (B) total comorbidity count in African American cancer survivors based on the occurrence of CIPN is provided. Patients with CIPN were more likely to have higher BMI and report more comorbidities. Both BMI and total comorbidities are divided into different categories, as indicated in the legend. Sample sizes for each group are indicated within each panel on the *x*‐axis

Several comorbidities were significantly or marginally associated with CIPN status. Notably, higher BMI at enrollment was positively associated with CIPN (covariate‐adjusted OR = 1.02 kg/m^2^, 95% CI: 1.006–1.04 kg/m^2^, *p* = 0.008), with 58.3% of obese patients reporting CIPN (Figure 1A). Depression (covariate‐adjusted OR = 1.62, 95% CI: 1.18–2.25, *p* = 0.003) and hypercholesterolemia (covariate‐adjusted OR = 1.32, 95% CI: 1.001–1.73, *p* = 0.05) were also more frequent in patients with CIPN. Both hypertension (covariate‐adjusted OR = 1.28, 95% CI: 0.98–1.67, *p* = 0.07) and diabetes (covariate‐adjusted OR = 1.33, 95% CI: 0.98–1.82, *p* = 0.06) were marginally associated with CIPN. Accordingly, patients with CIPN were more likely to report multiple comorbidities (covariate‐adjusted OR = 1.15, 95% CI: 1.07–1.24, *p* = 0.0001; Table 2; Figure 1B). Importantly, BMI at enrollment (*p* = 0.02) and depression (*p* = 0.004) remained statistically significant after including diabetes as a covariate in the model, indicating that these associations were independent of diabetes status, which has been shown to be highly correlated with these conditions.^20^, ^21^ By contrast, hypertension (*p* = 0.14) and hypercholesterolemia (*p* = 0.12) no longer remained statistically significant.

Analysis of sociodemographic factors revealed that CIPN was not more likely to occur in patients with less economic security. Specifically, there was no association between CIPN and the census tract poverty indicator (covariate‐adjusted OR = 1.04, 95% CI: 0.91–1.21, *p* = 0.54) or gross annual income (covariate‐adjusted OR = 0.96, 95% CI: 0.87–1.05, *p* = 0.36; Table 2).

### 3.3 Association with quality of life measures

**FIGURE 2:**
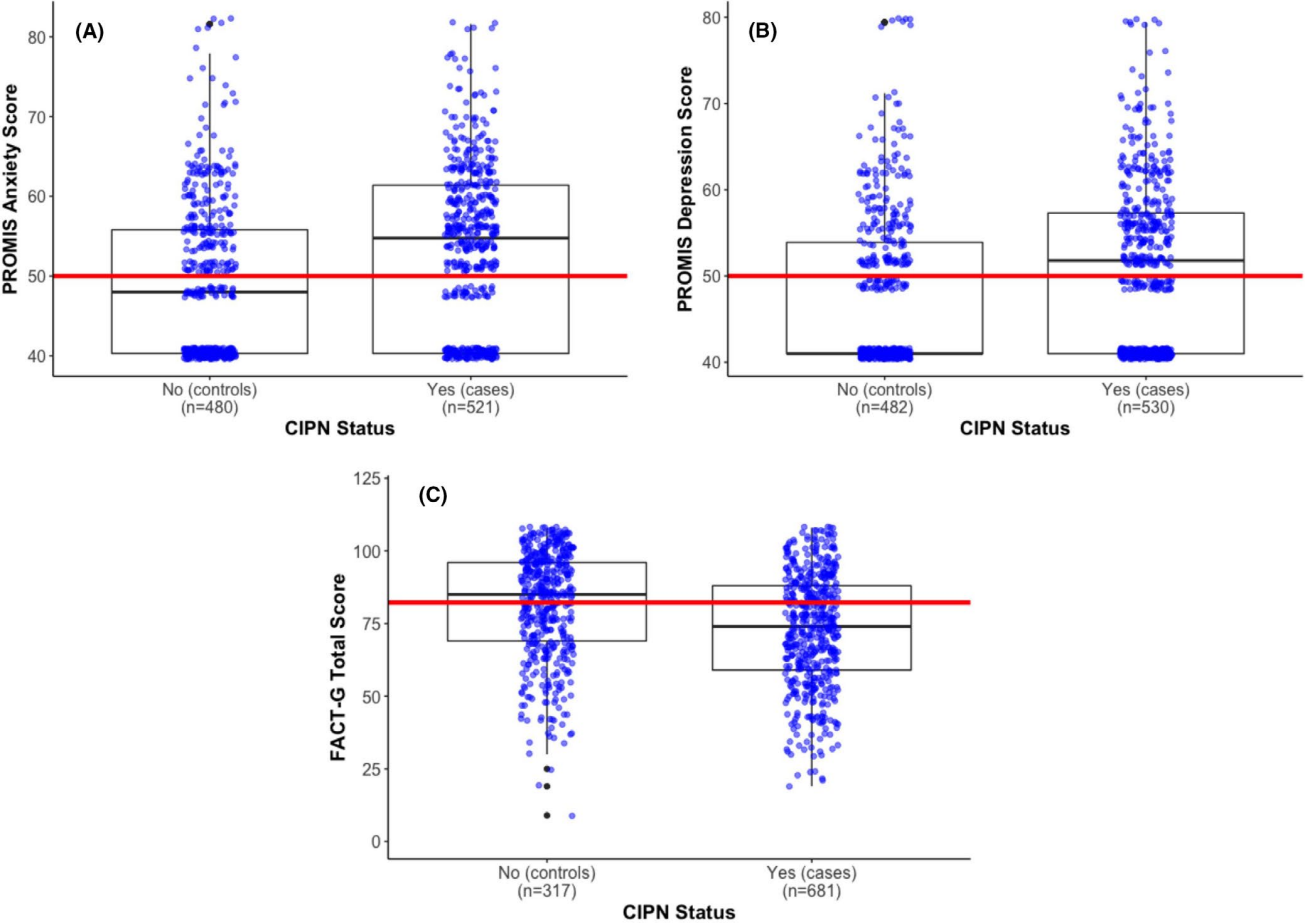
Effects of chemotherapy‐induced peripheral neuropathy (CIPN) status on quality of life and mood measures. The interquartile range of African American cancer survivors based on CIPN status is shown for the (A) PROMIS^®^ Anxiety, (B) PROMIS^®^ Depression, and (C) Functional Assessment of Cancer Therapy‐General (FACT‐G) total scales. Patients with CIPN are more likely to have higher PROMIS^®^ Anxiety and Depression scores, as well as a lower FACT‐G total score. The mean PROMIS^®^ Anxiety and Depression score (50) for the general US population and the mean FACT‐G total score (82.2) for elderly cancer patients are denoted by red lines. By contrast, mean PROMIS^®^ Anxiety and Depression scores in chemotherapy‐treated African American cancer survivors were 51.6 (*p* < 0.0001) and 49.5 (*p* = 0.10), respectively, while mean FACT‐G total score was 76.9 (*p* < 0.001). Deviation of the mean scores for African American cancer survivors from literature‐derived means was assessed using a one‐sample *t*‐test. Sample sizes for each group are indicated within each panel on the *x*‐axis

Using a linear regression model, we evaluated whether CIPN status was associated with several physical, mental, and emotional measures of quality of life. Patients with CIPN reported significantly higher PROMIS^®^ Anxiety (covariate‐adjusted *β* = 4.18, 95% CI: 2.92–5.45, *p* < 0.0001) and Depression (covariate‐adjusted *β* = 2.69, 95% CI: 1.53–3.84, *p* < 0.0001) scores (Table 3; Figure 2A,B), indicating worse mental health. Similarly, patients with CIPN reported significantly lower FACT‐G total scores (covariate‐adjusted *β* = −8.60, 95% CI: −10.88, −6.32, *p* < 0.0001; Table 3; Figure 2C), suggesting these patients’ overall health is worse than those who did not develop CIPN after completing chemotherapy. Accordingly, all FACT sub‐scores (FWB, PWB, SWB, and EWB) were significantly lower in CIPN cases than in controls (Table 3; Figure S1). These associations were very similar in breast cancer patients who made up approximately 50% of the patient cohort, except for the FACT SWB that was not statistically significant (Table S3). CIPN was significantly associated with FACT PWB in all cancer sites except prostate (Table S3). CIPN severity was also an important predictor of mood, as it was positively associated with PROMIS^®^ Anxiety (covariate‐adjusted *β* = 2.14, 95% CI: 1.34–2.94, *p* < 0.0001) and Depression (covariate‐adjusted *β* = 1.81, 95% CI: 1.07–2.55, *p* < 0.0001) scores (Table S4; Figure S2). FACT‐G total scores and all FACT sub‐scores were significantly lower in cases with moderate, moderate to severe, or severe CIPN than in cases with mild CIPN (Table S4; Figure S3).

**TABLE 3:** Association between quality of life and mood measures and chemotherapy‐induced peripheral neuropathy in African American cancer survivors

Clinical characteristic	*β* (95% CI)	*p* value	Covariate‐adjusted *β* (95% CI)	Covariate‐adjusted *p* value
PROMIS^®^ Anxiety score	4.39 (3.13, 5.64)	**<0.0001**	4.18 (2.92, 5.45)	**<0.0001**
PROMIS^®^ Depression score	3.05 (1.89, 4.20)	**<0.0001**	2.69 (1.53, 3.84)	**<0.0001**
FACT‐G total score	−8.70 (−11.07, −6.33)	**<0.0001**	−8.60 (−10.88, −6.32)	**<0.0001**
FACT FWB	−2.35 (−3.24, −1.46)	**<0.0001**	−2.40 (−3.29, −1.52)	**<0.0001**
FACT PWB	−4.01 (−4.81, −3.21)	**<0.0001**	−3.78 (−4.56, −3.01)	**<0.0001**
FACT SWB	−0.90 (−1.67, −0.14)	**0.02**	−0.98 (−1.75, −0.20)	**0.01**
FACT EWB	−1.74 (−2.34, −1.13)	**<0.0001**	−1.73 (−2.34, −1.12)	**<0.0001**

Note

Higher scores for PROMIS^®^ measures are indicative of more negative outcomes, while lower scores for FACT‐G measures are indicative of more negative outcomes.

Covariates included in the linear regression included age at diagnosis, sex, cancer site, BMI at enrollment, diabetes, hypertension, hypercholesterolemia, and total comorbidity count.

Bold indicates *p* ≤ 0.05.

Abbreviations: BMI, body mass index; EWB, emotional well‐being sub‐score; FACT, Functional Assessment of Cancer Therapy; FACT‐G, FACT (general); FWB, functional well‐being sub‐score; PROMIS^®^, Patient‐Reported Outcomes Measurement Information System; PWB, physical well‐being sub‐score; SWB, social well‐being sub‐scores.

## 4 DISCUSSION

The current study advances our understanding of demographic, clinical, and behavioral risk factors and comorbidities associated with CIPN in African American cancer survivors, a patient population not often examined in long‐term toxicity epidemiological studies. Our analyses revealed that females were more likely to report CIPN than males. Although sex differences for CIPN predisposition have previously been observed,^22^ it is highly likely that females in Detroit ROCS developed CIPN more frequently because they were more likely to receive CIPN‐inducing chemotherapy than men. Notably, approximately 50% of the patients in our cohort were diagnosed with breast cancer, a malignancy that is nearly always found in women, and often treated with CIPN‐inducing agents such as taxanes. The neurotoxic potential of taxanes is substantial, as they have been shown to increase CIPN risk 2.7‐fold when used as part of first‐line therapy in breast cancer patients.^23^ Furthermore, some of these patients receive carboplatin and/or docetaxel, which also increases CIPN risk.^24^ Although first‐line chemotherapy for metastatic prostate cancer includes docetaxel,^25^ only 4.4% of the cancer survivors included in this study were diagnosed with this malignancy. In addition, sex did not appear to be associated with CIPN in colorectal or lung cancer, which are treated with neurotoxic agents (oxaliplatin and cisplatin/carboplatin, respectively) in both male and female patients.

Neither alcohol consumption nor smoking was significantly associated with reported CIPN in African American cancer survivors. By contrast, both of these health behaviors were associated with increased risk for CIPN in non‐Hispanic White patients following cisplatin‐based chemotherapy.^3^, ^26^ The lack of an association between smoking status and CIPN in African Americans is particularly intriguing, as it was identified as a risk factor for CIPN in a meta‐analysis of 31 studies that predominantly included non‐Hispanic White patients.^2^ It has also been previously postulated that long‐term smoking based on number of pack‐years can increase susceptibility to paclitaxel‐induced neuropathy by reducing peripheral blood flow, likely due to increased nicotine exposure.^27^ Previous research shows that African Americans smoke fewer cigarettes^24^ and start smoking at an older age than non‐Hispanic Whites,^28^, ^29^ thus perhaps accounting for the lack of association in this sample. Notably, only 11.6% of patients (*n* = 118) in our cohort reported smoking one or more packs (≥20 cigarettes) per day, with only 25.4% (*n* = 30) of those individuals smoking more than one pack a day. In addition, the association of modifiable risk factors with CIPN may be treatment‐specific or dependent on the classification methods used to measure exposures. This is particularly evident for alcohol consumption, as a previous study of 169 patients administered oxaliplatin‐based chemotherapy identified high alcohol consumption (≥5 glasses in a single occasion for men and ≥4 glasses in a single occasion for women) to be a risk factor for CIPN.^30^ A similar association was found between excessive drinking (individuals who reported consuming ≥2 drinks/day on average in the past year) and cisplatin‐induced peripheral neuropathy.^3^ By contrast, two studies in breast cancer patients that dichotomized alcohol consumption based on whether the patient was drinking any amount of alcohol^31^ or whether they consumed more than one drink per month^32^ did not find an association with CIPN. In our cohort, the number of patients who consumed ≥5 drinks per week was relatively small (*n* = 96; 9.4%), indicating that most of the patients did not consume high amounts of alcohol. This lower level of alcohol consumption in African Americans may account for the lack of association between its use and CIPN in this study.

Nevertheless, several previously reported comorbidities associated with CIPN were found to be statistically or marginally significant in this study of African American cancer survivors, including BMI, hypertension, hypercholesterolemia, depression, and diabetes. The CIPN patients were also more likely to report a greater number of comorbidities than controls. The association of CIPN with BMI was particularly prominent, as 58.3% of obese participants treated with chemotherapy reported having CIPN versus just 48.3% of participants with normal BMI. Obesity is a common risk factor for hypertension, hypercholesterolemia, and diabetes, yet BMI and hypercholesterolemia were independent risk factors for CIPN.^33^ Hypertension and hypercholesterolemia have been previously reported as independent risk factors for CIPN in non‐Hispanic White testicular cancer survivors.^3^ Diabetes has also been shown to be strongly associated with CIPN, with diabetic cancer survivors having two times the odds of developing neuropathy.^12^ By contrast, none of the diabetic survivors in that same study (those who did not receive taxane or platinum‐based therapy; *n* = 59) developed neuropathy, indicating that the neuropathy observed in taxane‐treated diabetic survivors likely developed during the course of therapy. In addition to the diabetic neuropathy cancer survivors may experience, it has been shown that diabetic survivors receiving taxane‐based chemotherapy are much more likely to develop neuropathy than those who received other forms of chemotherapy^12^ and that individuals receiving a taxane have an increased susceptibility to developing hyperglycemia.^34^

Although CIPN status was not associated with moderate physical activity, African American survivors with CIPN were far less likely to report vigorous physical activity. It cannot be determined whether vigorous activity decreases the risk of CIPN development or if African American survivors who develop CIPN can no longer engage in vigorous exercise. The lack of exercise further increases the susceptibility to obesity and increases the risk of several comorbidities that can compromise the overall quality of life. In addition, it has been demonstrated that exercise can alleviate and/or reduce the overall severity of CIPN that patients experience,^35^, ^36^ indicating that individuals who are unable to engage in vigorous exercise may be at risk of exacerbating symptoms.

Due to strong associations between CIPN and multiple comorbidities, and the resulting functional limitations caused by poorer health, we evaluated whether CIPN was associated with poorer quality of life and lower mood. Using the PROMIS^®^ scales for Anxiety and Depression, we found that African American survivors with CIPN were much more likely to report greater anxiety and depression than survivors without CIPN in this sample. This is consistent with previous research that shows that non‐Hispanic White survivors with high CIPN scores reported more anxiety and depressive symptoms and more fatigue.^37^ Among survivors with high CIPN in that study (upper 30% of CIPN scores from linearly transformed EORTC QLQ‐CIPN20 scale), those who were anxious and/or depressed reported more fatigue compared with those without psychological distress. Importantly, many of these associations remained statistically significant when examined across different cancer types, indicating that they were not driven solely by breast cancer patients who made up approximately half of the cohort. This suggests that regardless of their diagnosis or treatment regimen, patients who develop CIPN are more likely to experience mental health issues following the completion of therapy. Furthermore, these findings suggest that the mental health of cancer survivors is tightly linked to physical health. As survivors with CIPN are already prone to reduced mobility due to pain experienced in the upper and lower extremities, increased fatigue would further reduce physical health, thereby increasing or exacerbating obesity and other associated comorbidities. African American survivors in our study with CIPN reported having an overall poorer quality of life that affected their physical, emotional, social, and functional well‐being, as indicated by their lower overall FACT‐G score and subscale scores, respectively. Although there were some differences in QOL and mood across different cancer sites, overall our results show that CIPN is more prevalent in African American survivors with multiple comorbidities and results in reduced quality of life. It is important to note that CIPN severity may also serve as a predictor of overall quality of life and mood in patients following chemotherapy, as African American survivors with more severe CIPN reported higher PROMIS^®^ scores and lower FACT‐G scores than those with mild CIPN. Consequently, African American survivors with moderate to severe CIPN appear particularly susceptible to reduced quality of life, and screening these individuals in follow‐up visits to evaluate their physical and mental health is warranted.

Major strengths of our study include the comprehensiveness of the Detroit ROCS questionnaire allowing evaluation of predictors of CIPN and quality of life as measured by several domains of overall health. Using an African American cohort of cancer survivors, we focused exclusively on a patient population that is understudied and remains poorly characterized. To our knowledge, it also marks the largest study of CIPN in African American cancer survivors to date and provides novel insight regarding potential differences in risk factors in a population‐based analysis, making the results more generalizable. One limitation of our study is the lack of detailed treatment drug, dose, and duration data on patients. The Detroit ROCS dataset was not designed specifically to capture clinical toxicity‐related data. Although we can make assumptions as to the agents used for specific cancer sites (i.e., taxanes for breast cancer and platinating agents for lung cancer), we are unable to stratify the analyses for specific drugs or dosage. We relied on patient‐reported outcomes to define CIPN. Ideally, our analysis would have reported associations with both patient‐reported and physician‐graded CIPN, as it has been previously demonstrated that clinician‐rated scores can diverge from patients’ perception.^38^ However, it is important to note that an NCI Clinical Trials Planning Meeting concluded that patient‐reported CIPN may be superior to physical exam and can be considered an effective definition of the toxicity.^39^ Patient‐reported outcomes also serve as the gold standard for understanding how patients are feeling (i.e., quality of life and feelings of anxiety and depression). Another limitation is that the data are cross‐sectional such that survivors reported risk factor and quality of life data at the same time as reports of CIPN. Thus, we cannot determine temporality of higher anxiety and depression or lower quality of life relative to their cancer diagnosis, chemotherapy treatment, or post‐treatment. Future research may benefit from examining these relationships over time to better understand causality. However, we do know that if mental health and quality of life changes were driven solely by cancer diagnosis or receipt of chemotherapy, we would not see associations with CIPN because the control group also had cancer diagnoses and received chemotherapy. It is also possible having relatively small sample sizes for some cancer types (i.e., endometrial and prostate) masked some associations that may have been identified with larger sample sizes. Finally, it should be noted that several risk factors previously identified for CIPN have been inconsistent across studies focused predominantly on non‐Hispanic whites, including smoking, alcohol consumption, age, and diabetes status. This discrepancy may be due in part to the variability in defining CIPN as a phenotype for investigation, and should be considered for future studies that seek to determine risk factors for CIPN in African American cancer survivors.

This study demonstrates the critical importance of examining underrepresented patient populations for common toxicities experienced following chemotherapy. We identified demographic, clinical, and behavioral risk factors for CIPN that vary from the literature by race. Furthermore, African American cancer survivors with CIPN reported poorer quality of life that should be studied further. In view of our results, healthcare providers can improve the management of African American cancer survivors by assessing factors related to their risk for CIPN (e.g., comorbidities and health behaviors). To build on the results of this study, future research will benefit from exploring the effects of other common chemotherapy‐induced toxicities in African American cancer survivors that may underlie racial health disparities in treatment and care to support more equitable delivery of quality healthcare.

## CONFLICT OF INTEREST

The authors do not have any conflict of interest.

## INFORMED CONSENT

The Karmanos Cancer Institute Protocol and Monitoring Review Board and the Wayne State University Institutional Review Board (#050417M1F) reviewed and approved this research with all participants in the Detroit ROCS study providing informed consent.

## Supporting information

Supplementary Material

## Data Availability

The data that support the findings of this study are available from the corresponding author upon reasonable request.
