# Supplementary material for: Chemotherapy‐induced peripheral neuropathy in African American cancer survivors: Risk factors and quality of life outcomes
Source: Cancer Med. 2021 Oct 23;10(22):8151–61. doi: 10.1002/cam4.4328 (PMC8607253; doi:10.1002/cam4.4328)
Supplement: Supplementary file 1 — Supplementary Material [file CAM4-10-8151-s001.docx]

Supplemental Materials to:

**Chemotherapy-Induced Peripheral Neuropathy in African American Cancer Survivors: Risk Factors and Quality of Life Outcomes**

Matthew R. Trendowski^1^, Christine M. Lusk^1,2^, Julie J. Ruterbusch^1,2^, Randell Seaton^1,2^, Michael S. Simon^1,2^, Mark K. Greenwald^3^, Felicity W.K. Harper^1,2^, Jennifer L. Beebe-Dimmer^1,2^, and Ann G. Schwartz^1,2, *^

^1^Wayne State University School of Medicine, Department of Oncology, 4100 John R., Detroit, MI, USA; ^2^Karmanos Cancer Institute, 4100 John R., Detroit, MI, USA; ^3^Wayne State University School of Medicine, Department of Psychiatry and Behavioral Neurosciences, 3901 Chrysler Service Drive, Suite 2A, Detroit, MI, USA


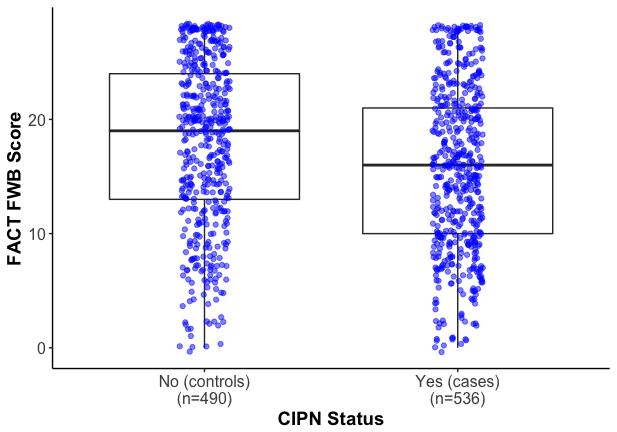

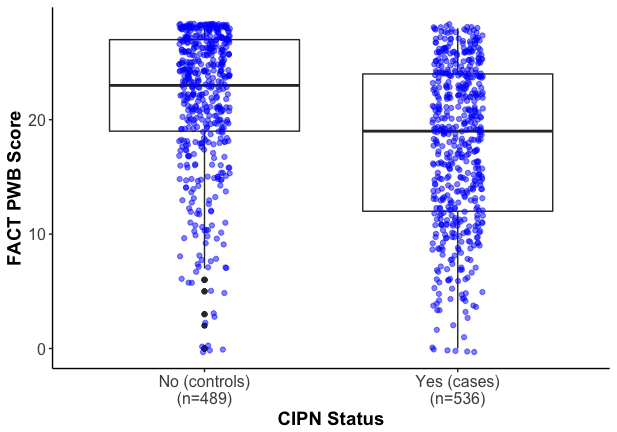


D

C

B

A


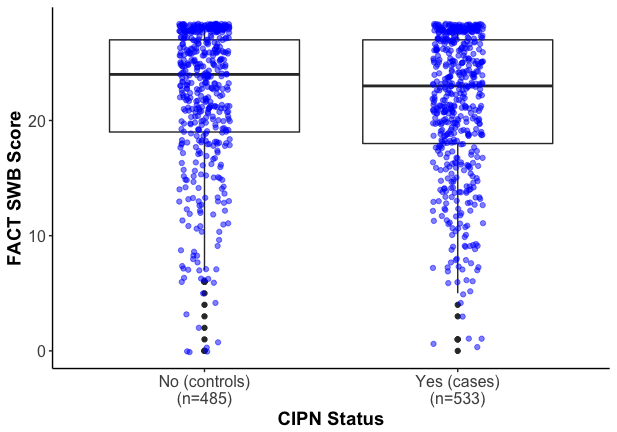

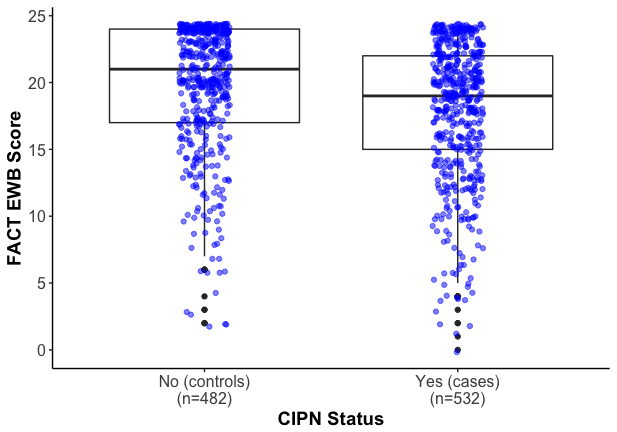


**Supplemental Figure 1. Effects of CIPN Status on FACT-G Subscores.** The interquartile range of African American cancer survivors based on CIPN status is shown for the **A)** FWB, **B)** PWB, **C)** SWB, and **D)** EWB FACT-G subscores. Patients with CIPN are more likely to have lower FWB, PWB, SWB, and EWB subscores, indicative of worse functional, physical, social, and emotional well-being. Sample sizes for each group are indicated within each panel on the x-axis.

**
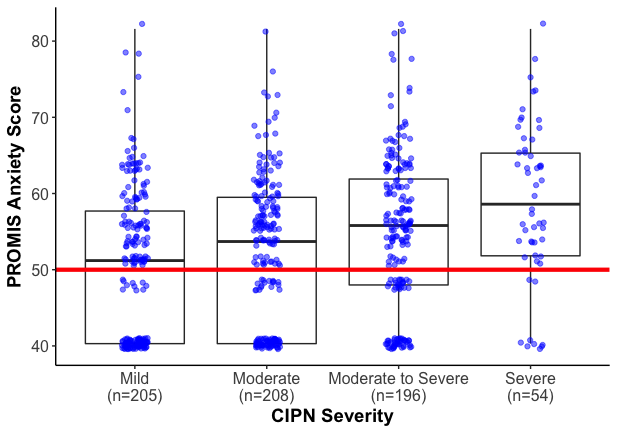
** **
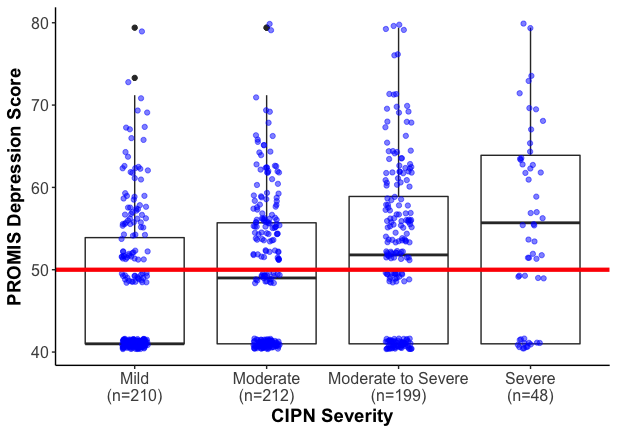
**

B

A

C

**
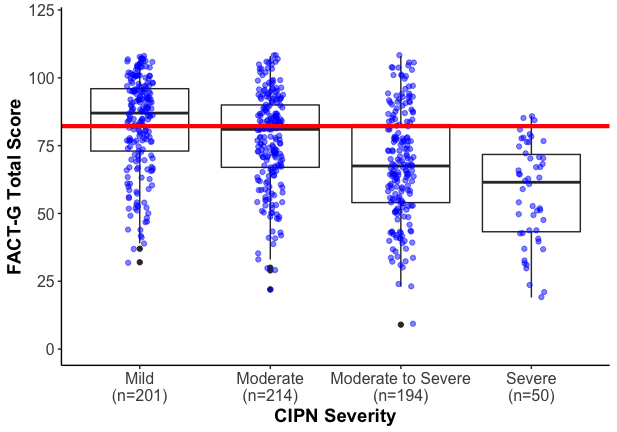
**

**Supplemental Figure 2. Effects of CIPN Severity on Quality of Life and Mood Measures.** The interquartile range of African American cancer survivors based on CIPN severity is shown for the **A)** PROMIS^®^ Anxiety, **B)** PROMIS^®^ Depression, and **C)** FACT-G total scales. Patients with moderate, moderate to severe, or severe CIPN are more likely to have higher PROMIS^®^ Anxiety and Depression scores, as well as a lower FACT-G total score than patients with mild CIPN. The mean PROMIS^®^ Anxiety and Depression score (50) for the general U.S. population and the mean FACT-G total score (82.2) for elderly cancer patients are denoted by red lines. Sample sizes for each group are indicated within each panel on the x-axis.


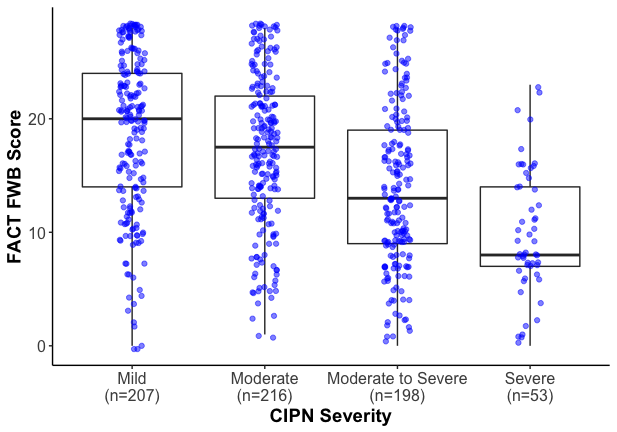

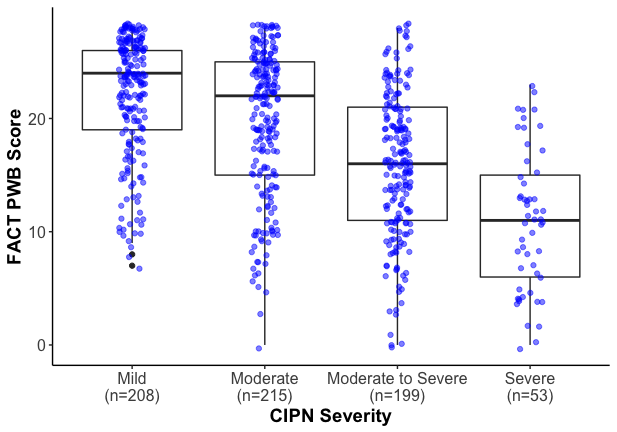


B

A

D

C


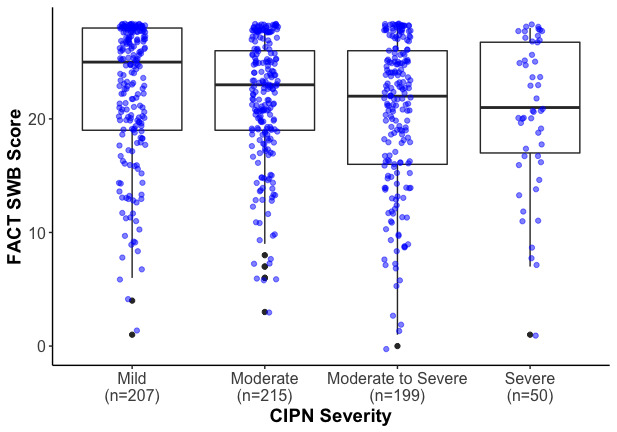

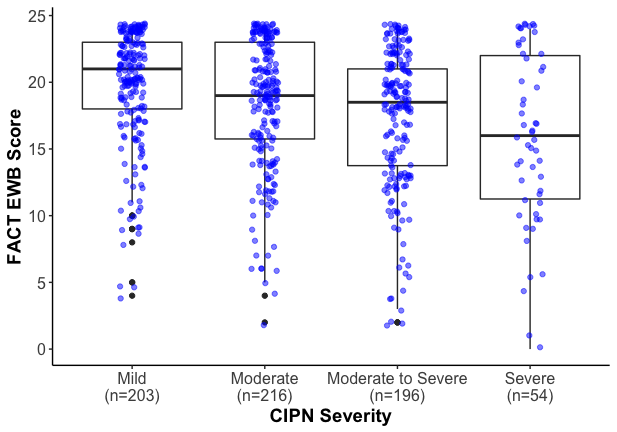


**Supplemental Figure 3. Effects of CIPN Severity on FACT-G Subscores.** The interquartile range of African American cancer survivors based on CIPN severity is shown for the **A)** FWB, **B)** PWB, **C)** SWB, and **D)** EWB FACT-G subscores. Patients with moderate, moderate to severe, and severe CIPN are more likely to have lower FWB, PWB, SWB, and EWB subscores than patients with mild CIPN, indicative of worse functional, physical, social, and emotional well-being. Sample sizes for each group are indicated within each panel on the x-axis.

**Supplemental Table 1. Additional Clinical and Sociodemographic Characteristics for African American Cancer Survivors Treated with Chemotherapy by Reported Chemotherapy-Induced Peripheral Neuropathy Status.**

| **Characteristic** | **All Patients** | **CIPN: No**  **(Controls)** | **CIPN: Yes**  **Cases** |
| --- | --- | --- | --- |
| **n** | 1,045 | 495 | 550 |
| **Age at Survey (years)** |  |  |  |
| Median (range) | 59 (25-84) | 60 (26-84) | 58 (25-84) |
| Under 50 | 244 (23.3%) | 116 (23.4%) | 128 (23.3%) |
| 50+ | 801 (76.7%) | 379 (76.6%) | 422 (76.7%) |
| **Surgical Resection of Tumor^a^** |  |  |  |
| No | 256 (24.7%) | 150 (30.5%) | 106 (19.4%) |
| Yes | 780 (75.3%) | 341 (69.4%) | 439 (80.6%) |
| I**mmunotherapy^b^** |  |  |  |
| No | 914 (91.3%) | 427 (89.5%) | 487 (92.9%) |
| Yes | 87 (8.7%) | 50 (10.5%) | 37 (7.1%) |
| **Radiotherapy^c^** |  |  |  |
| No | 407 (39.4%) | 181 (36.9%) | 226 (41.5%) |
| Yes | 627 (60.6%) | 309 (63.1%) | 318 (58.5%) |
| **Hormone Therapy^d^** |  |  |  |
| No | 848 (83.1%) | 403 (83.1%) | 445 (83.2%) |
| Yes | 172 (16.9%) | 82 (16.9%) | 90 (16.8%) |
| **Ever Smoked (100 Cigarettes)^e^** |  |  |  |
| No | 534 (51.6%) | 244 (49.8%) | 290 (53.3%) |
| Yes | 500 (48.4%) | 246 (50.2%) | 254 (46.7%) |
| **Current Smoker^f^** |  |  |  |
| No | 879 (85.2%) | 420 (85.7%) | 459 (84.7%) |
| Yes | 153 (14.8%) | 70 (14.3%) | 83 (15.3%) |
| **Number of Cigarettes Smoked per Day^g^** |  |  |  |
| None | 539 (53.1%) | 247 (51.1%) | 292 (54.9%) |
| 1-9 | 225 (22.2%) | 102 (21.1%) | 123 (23.1%) |
| 10-19 | 133 (13.1%) | 69 (14.3%) | 64 (12.0%) |
| ≥ 20 | 118 (11.6%) | 65 (13.5%) | 53 (10.0%) |
| **Alcohol Consumption^h^** |  |  |  |
| No | 630 (60.6%) | 292 (59.2%) | 338 (61.8%) |
| Yes | 410 (39.4%) | 201 (40.8%) | 209 (38.2%) |
| **Number of Drinks per Week^i^** |  |  |  |
| None | 682 (66.6%) | 317 (65.0%) | 365 (68.1%) |
| 1 | 92 (9.0%) | 47 (9.6%) | 45 (8.4%) |
| 2 | 74 (7.2%) | 38 (7.8%) | 36 (6.7%) |
| 3 | 42 (4.1%) | 20 (4.1%) | 22 (4.1%) |
| 4 | 38 (3.7%) | 15 (3.1%) | 23 (4.3%) |
| ≥ 5 | 96 (9.4%) | 51 (10.5%) | 45 (8.4%) |
| **Any Physical Activity^j^** |  |  |  |
| No | 370 (35.6%) | 182 (36.9%) | 188 (34.4%) |
| Yes | 669 (64.4%) | 311 (63.1%) | 358 (65.6%) |
| **Moderate Physical Activity^k^** |  |  |  |
| No | 490 (47.3%) | 237 (48.4%) | 253 (46.4%) |
| Yes | 545 (52.7%) | 253 (51.6%) | 292 (53.6%) |
| **Vigorous Physical Activity^l^** |  |  |  |
| No | 740 (71.4%) | 335 (68.0%) | 405 (74.4%) |
| Yes | 297 (28.6%) | 158 (32.0%) | 139 (25.6%) |
| **Hypertension^m^** |  |  |  |
| No | 427 (41.4%) | 213 (43.9%) | 214 (39.1%) |
| Yes | 605 (58.6%) | 272 (56.1%) | 333 (60.9%) |
| **Hypercholesterolemia^n^** |  |  |  |
| No | 669 (65.1%) | 324 (67.2%) | 345 (63.3%) |
| Yes | 358 (34.9%) | 158 (32.8%) | 200 (36.7%) |
| **History of Depression^o^** |  |  |  |
| No | 821 (79.8%) | 409 (84.0%) | 412 (76.0%) |
| Yes | 208 (20.2%) | 78 (16.0%) | 130 (24.0%) |
| **Bone Fracture after 50^p^** |  |  |  |
| No | 975 (93.9%) | 465 (95.1%) | 510 (92.9%) |
| Yes | 63 (6.1%) | 24 (4.9%) | 39 (7.1%) |
| **Diabetes^q^** |  |  |  |
| No | 805 (78.0%) | 389 (80.2%) | 416 (76.1%) |
| Yes | 227 (22.0%) | 96 (19.8%) | 131 (23.9%) |
| **Census Tract Poverty Indicator^r^** |  |  |  |
| 0-4.9% | 52 (5.0%) | 22 (4.5%) | 30 (5.5%) |
| 5-9.9% | 128 (12.3%) | 66 (13.4%) | 62 (11.3%) |
| 10-19.9% | 215 (20.7%) | 97 (19.7%) | 118 (21.6%) |
| 20-100% | 644 (62.0%) | 307 (62.4%) | 337 (61.6%) |
| **Education^s^** |  |  |  |
| Less than High School | 95 (9.2%) | 45 (9.2%) | 50 (9.2%) |
| High School/GED | 263 (25.5%) | 133 (27.2%) | 130 (24.0%) |
| Some College | 278 (27.0%) | 126 (25.8%) | 152 (28.0%) |
| 2-Year College Degree | 131 (12.7%) | 55 (11.3%) | 76 (14.0%) |
| 4-Year College Degree | 112 (10.9%) | 54 (11.1%) | 58 (10.7%) |
| Graduate/Professional Degree | 151 (14.7%) | 75 (15.4%) | 76 (14.0%) |
| **Marital Status^t^** |  |  |  |
| Married | 316 (30.4%) | 156 (31.8%) | 160 (29.2%) |
| Living with Partner in a Marriage-Like Relationship | 48 (4.6%) | 22 (4.5%) | 26 (4.7%) |
| Widowed | 105 (10.1%) | 53 (10.8%) | 52 (9.5%) |
| Divorced | 217 (20.9%) | 90 (18.4%) | 127 (23.2%) |
| Separated | 46 (4.4%) | 11 (2.2%) | 35 (6.4%) |
| Never Married | 305 (29.4%) | 158 (32.2%) | 147 (26.8%) |
| Other | 1 (0.1%) | 0 (0%) | 1 (0.2%) |

Abbreviation: GED: general educational development

^a^9 patients did not report whether their tumor was surgically resected.

^b^44 patients did not report whether they received immunotherapy.

^c^11 patients did not report whether they received radiotherapy.

^d^25 patients did not report whether they received hormone therapy.

^e^11 patients did not report whether they ever smoked (100 cigarettes).

^f^13 patients did not report whether they currently smoke.

^g^30 patients did not report number of cigarettes smoked per day.

^h^5 patients did not report whether they consume alcohol.

^i^21 patients did not report number of drinks consumed per week.

^j^6 patients did not report whether they have any form of physical activity.

^k^10 patients did not report whether they engage in moderate physical activity.

^l^10 patients did not report whether they engage in vigorous physical activity.

^m^13 patients did not report hypertension status.

^n^18 patients did not report hypercholesterolemia status.

^o^16 patients did not report depression status.

^p^7 patients did not report bone fracture after 50 status.

^q^13 patients did not report diabetes status.

^r^6 patients did not report poverty status.

^s^15 patients did not report level of education.

^t^7 patients did not report marital status.

**Supplemental Table 2. Identification of Clinical Characteristics Associated with CIPN in African American Cancer Survivors.**

| **Clinical Characteristic** | **OR (95% CI)** | **p-value** |
| --- | --- | --- |
| **Sex** | 1.57 (1.19, 2.10) | **0.001** |
| **Cancer Site^a^** |  |  |
| Breast | 1.00 (Ref) |  |
| Colorectal | 1.29 (0.88, 1.93) | 0.21 |
| Endometrial | 2.32 (1.04, 6.24) | 0.06 |
| Lung | 0.29 (0.20, 0.42) | **<0.0001** |
| Prostate | 0.21 (0.11, 0.39) | **<0.0001** |
| Other^b^ | 0.70 (0.43, 1.19) | 0.18 |

^a^Breast cancer patients were used as the reference group.

^b^Other cancers were only included for individuals diagnosed before age 50.

Bold indicates p≤0.05.

**Supplemental Table 3. Association Between Quality of Life and Mood Measures and CIPN in African American Cancer Survivors Based on Cancer Site.**

| **Clinical Characteristic** | **β (95% CI)** | **p-value** | **Covariate-Adjusted β (95% CI)** | **Covariate- Adjusted p-value** |
| --- | --- | --- | --- | --- |
| **Breast (n=511)** | | | | |
| PROMIS^®^ Anxiety Score | 5.39 (3.64, 7.14) | **<0.0001** | 4.82 (3,12, 6.53) | **<0.0001** |
| PROMIS^®^ Depression Score | 4.14 (2.57, 5.72) | **<0.0001** | 3.47 (1.94, 5.01) | **<0.0001** |
| FACT-G Total Score | -10.21 (-13.40, -7.01) | **<0.0001** | -7.93 (-10.93, -4.95) | **<0.0001** |
| FACT FWB | -3.26 (-4.48, -2.04) | **<0.0001** | -2.52 (-3.72, -1.32) | **<0.0001** |
| FACT PWB | -4.01 (-5.20, -3.01) | **<0.0001** | -3.38 (-4.40, -2.36) | **<0.0001** |
| FACT SWB | -0.94 (1.97, 0.10) | 0.07 | -0.59 (-1.51, -0.54) | 0.26 |
| FACT EWB | -2.09 (-2.88, -1.30) | **<0.0001** | -1.71 (-2.48, -0.95) | **<0.0001** |
| **Colorectal (n=191)** | | | | |
| PROMIS^®^ Anxiety Score | 3.07 (0.18, 5.95) | **0.04** | 2.64 (-0.34, 5.62) | 0.08 |
| PROMIS^®^ Depression Score | 1.11 (-1.77, 3.98) | 0.45 | 0.93 (-1.94, 3.79) | 0.52 |
| FACT-G Total Score | -9.61 (-15.43, -3.79) | **0.001** | -9.58 (-15.32, -3.83) | **0.001** |
| FACT FWB | -2.60 (-4.78, -0.41) | **0.02** | -2.41 (-4.64, -0.18) | **0.03** |
| FACT PWB | -4.64 (-6.65, -2.62) | **<0.0001** | -4.80 (-6.82, -2.78) | **<0.0001** |
| FACT SWB | -0.49 (-2.39, 1.41) | 0.61 | -0.80 (-2.73, 1.13) | 0.41 |
| FACT EWB | -1.92 (-3.36, -0.48) | **0.009** | -1.58 (-3.06, -0.11) | **0.04** |
| **Endometrial (n=45)** | | | | |
| PROMIS^®^ Anxiety Score | 3.38 (-2.20, 8.96) | 0.23 | 3.22 (-2.76, 9.21) | 0.28 |
| PROMIS^®^ Depression Score | 0.92 (-4.52, 6.46) | 0.74 | -0.23 (-6.01, 5.55) | 0.94 |
| FACT-G Total Score | -7.89 (-20.30, 4.52) | 0.21 | -5.56 (-19.50, 8.37) | 0.42 |
| FACT FWB | -2.87 (-7.48, 1.73) | 0.22 | -3.03 (-8.10, 2.05) | 0.23 |
| FACT PWB | -3.31(-7.44, 0.82) | 0.11 | -4.41 (-8.07, -0.76) | **0.02** |
| FACT SWB | -1.16 (-5.26, 2.94) | 0.57 | -0.78 (-5.39, 3.82) | 0.73 |
| FACT EWB | -1.50 (-4.44, 1.44) | 0.31 | -1.29 (-4.45, 1.87) | 0.41 |
| **Lung (n=158)** | | | | |
| PROMIS^®^ Anxiety Score | 4.04 (0.30, 7.78) | **0.03** | 3.33 (-0.64, 7.29) | 0.10 |
| PROMIS^®^ Depression Score | 4.11 (0.88, 7.34) | **0.01** | 3.10 (-0.33, 6.52) | 0.08 |
| FACT-G Total Score | -9.34 (-16.16, -2.51) | **0.008** | -6.91 (-13.75, -0.06) | **0.05** |
| FACT FWB | -2.18 (-4.71, 0.36) | 0.09 | -1.42 (-3.95, 1.10) | 0.27 |
| FACT PWB | -4.12 (-6.33, -1.92) | **0.0003** | -3.11 (-5.26, -0.96) | **0.005** |
| FACT SWB | -1.84 (-4.05, 0.37) | 0.10 | -1.69 (-4.03, 0,65) | 0.16 |
| FACT EWB | -1.79 (-3.82, 0.22) | 0.08 | -1.01 (-3.13, 1.11) | 0.35 |
| **Prostate (n=46)** | | | | |
| PROMIS^®^ Anxiety Score | 5.21 (-1.61, 12.03) | 0.13 | 5.93 (-1.64, 13.50) | 0.12 |
| PROMIS^®^ Depression Score | 2.46 (-4.68, 9.60) | 0.49 | 3.79 (-3.70, 11.28) | 0.31 |
| FACT-G Total Score | -9.25 (-21.81, 3.31) | 0.15 | -8.89 (-22.18, 4.40) | 0.18 |
| FACT FWB | -3.14 (-7.84, 1.57) | 0.19 | -4.01 (-9.19, 1.18) | 0.13 |
| FACT PWB | -3.52 (-7.87, 0.84) | 0.11 | -3.88 (-8.21, 0.45) | 0.08 |
| FACT SWB | -2.54 (-7.52, 2.45) | 0.31 | -1.86 (-7.42, 3.50) | 0.50 |
| FACT EWB | -1.82 (-4.42, -0.78) | 0.17 | -2.07 (-5.08, 0.95) | 0.17 |
| **Other^a^ (n=76)** | | | | |
| PROMIS^®^ Anxiety Score | 3.61 (-1.93, 9.14) | 0.20 | 3.82 (-2.07, 9.71) | 0.20 |
| PROMIS^®^ Depression Score | 3.15 (-1.95, 8.24) | 0.23 | 4.21 (-1.36, 9.78) | 0.14 |
| FACT-G Total Score | -13.21 (-23.44, -2.97) | **0.01** | -11.56 (-21.63, -1.48) | **0.03** |
| FACT FWB | -2.47 (-6.23, 1.28) | 0.19 | -1.78 (-5.57, 2.00) | 0.35 |
| FACT PWB | -5.66 (-9.10, -2.22) | **0.001** | -5.12 (-8.42, -1.81) | **0.003** |
| FACT SWB | -2.90 (-5.86, 0.07) | 0.06 | -2.26 (-5.29, 0.86) | 0.15 |
| FACT EWB | -2.22 (-4.99, 0.56) | 0.12 | -2.46 (-5.37, 0.46) | 0.10 |

^a^Other cancers were only included for individuals diagnosed before age 50.

Abbreviations: EWB: emotional well-being sub-score; FACT: Functional Assessment of Cancer Therapy; FACT-G: FACT (general); FWB: functional well-being sub-score; PROMIS: Patient-Reported Outcomes Measurement Information System; PWB: physical well-being sub-score; SWB: social well-being sub-score. Covariates included in the linear regression included age at diagnosis, sex, current BMI, diabetes, hypertension, hypercholesterolemia, and total comorbidity count.

Analyses of breast, endometrial, and prostate cancer did not include sex as a covariate.

Bold indicates p≤0.05.

**Supplemental Table 4. Association Between Quality of Life Measures and CIPN Severity in African American Cancer Survivors.**

| **Clinical Characteristic** | **β (95% CI)** | **p-value** | **Covariate-Adjusted β (95% CI)** | **Covariate- Adjusted p-value** |
| --- | --- | --- | --- | --- |
| PROMIS^®^ Anxiety Score | 2.52 (1.73, 3.31) | **<0.0001** | 2.14 (1.34, 2.94) | **<0.0001** |
| PROMIS^®^ Depression Score | 2.34 (1.60, 3.08) | **<0.0001** | 1.81 (1.07, 2.55) | **<0.0001** |
| FACT-G Total Score | -8.12 (-9.61, -6.63) | **<0.0001** | -7.04 (-8.47, -5.61) | **<0.0001** |
| FACT FWB | -2.66 (-3.21, -2.11) | **<0.0001** | -2.34 (-2.89, -1.79) | **<0.0001** |
| FACT PWB | -3.41 (-3.90, -2.93) | **<0.0001** | -3.07 (-3.54, -2.60) | **<0.0001** |
| FACT SWB | -0.80 (-1.29, -0.31) | **0.001** | -0.64 (-1.13, -0.15) | **0.01** |
| FACT EWB | -1.20 (-1.60, -0.80) | **<0.0001** | -0.97 (-1.37, -0.57) | **<0.0001** |

Abbreviations: EWB: emotional well-being sub-score; FACT: Functional Assessment of Cancer Therapy; FACT-G: FACT (general); FWB: functional well-being sub-score; PROMIS^®^: Patient-Reported Outcomes Measurement Information System; PWB: physical well-being sub-score; SWB: social well-being subscore.

Covariates included in the linear regression included age at diagnosis, sex, cancer site, current BMI, diabetes, hypertension, hypercholesterolemia, and total comorbidity count.

Bold indicates p≤0.05.
